# Application of graft-derived cell-free DNA for solid organ transplantation

**DOI:** 10.3389/fimmu.2024.1461480

**Published:** 2024-09-23

**Authors:** Wenqiang Zhang, Bin Liu, Dan Jia, Ruiyu Wang, Hongliang Cao, Hao Wu, Zihao Ye, Baoshan Gao

**Affiliations:** Department of Urology II, The First Hospital of Jilin University, Changchun, China

**Keywords:** graft-derived cell-free DNA, gd-cfDNA, organ transplantation, graft injury, rejection, non-invasive biomarker

## Abstract

Monitoring the status of grafts and the occurrence of postoperative complications, such as rejection, is crucial for ensuring the success and long-term survival of organ transplants. Traditional histopathological examination, though effective, is an invasive procedure and poses risks of complications, making frequent use impractical. In recent years, graft-derived cell-free DNA (gd-cfDNA) has emerged as a promising non-invasive biomarker. It not only provides early warnings of rejection and other types of graft injury but also offers important information about the effectiveness of immunosuppressive therapy and prognosis. gd-cfDNA shows potential in the monitoring of organ transplants. The early, real-time information on graft injury provided by gd-cfDNA facilitates timely individualized treatment and improves patient outcomes. However, the progress of research on gd-cfDNA varies across different organs. Therefore, this article will comprehensively review the application and findings of gd-cfDNA in monitoring various solid organs, discussing the advantages, limitations, and some future research directions to aid in its clinical application.

## 1 Introduction

Solid organ transplantation is a crucial treatment for end-stage organ failure. However, post-transplantation, there is a risk of occurrence of transplant-related complications (such as rejection) and graft function decline, which may lead to graft loss and reduced patient survival rates (1). Therefore, monitoring the status of transplanted organs is vital for the long-term survival of recipients. Only more comprehensive monitoring of the grafts can reduce the happening of adverse outcomes and improve patient survival rates. The current gold standard for assessing graft health remains histopathological biopsy. However, due to its invasive nature, high cost, and associated risk of complications, its clinical application is limited, making it unsuitable for frequent routine monitoring (2, 3). Other indicators for monitoring organ function, such as serum creatinine for kidneys and transaminases for the liver, have low sensitivity to graft injury and exhibit a lag in response (4). Thus, there is currently a lack of reliable, low-risk, and suitable methods for repeated monitoring. In recent years, graft-derived cell-free DNA (gd-cfDNA), released by damaged graft cells, has emerged as a novel non-invasive biomarker for monitoring post-transplantation rejection and other types of graft injury, providing a new option for continuous, minimally invasive monitoring of organ transplants (5). However, the application of gd-cfDNA is still in the early exploratory stages and has not yet reached a consensus. This article will comprehensively review the current research developments across various types of transplanted organs.

## 2 Production of gd-cfDNA

**Figure 1:**
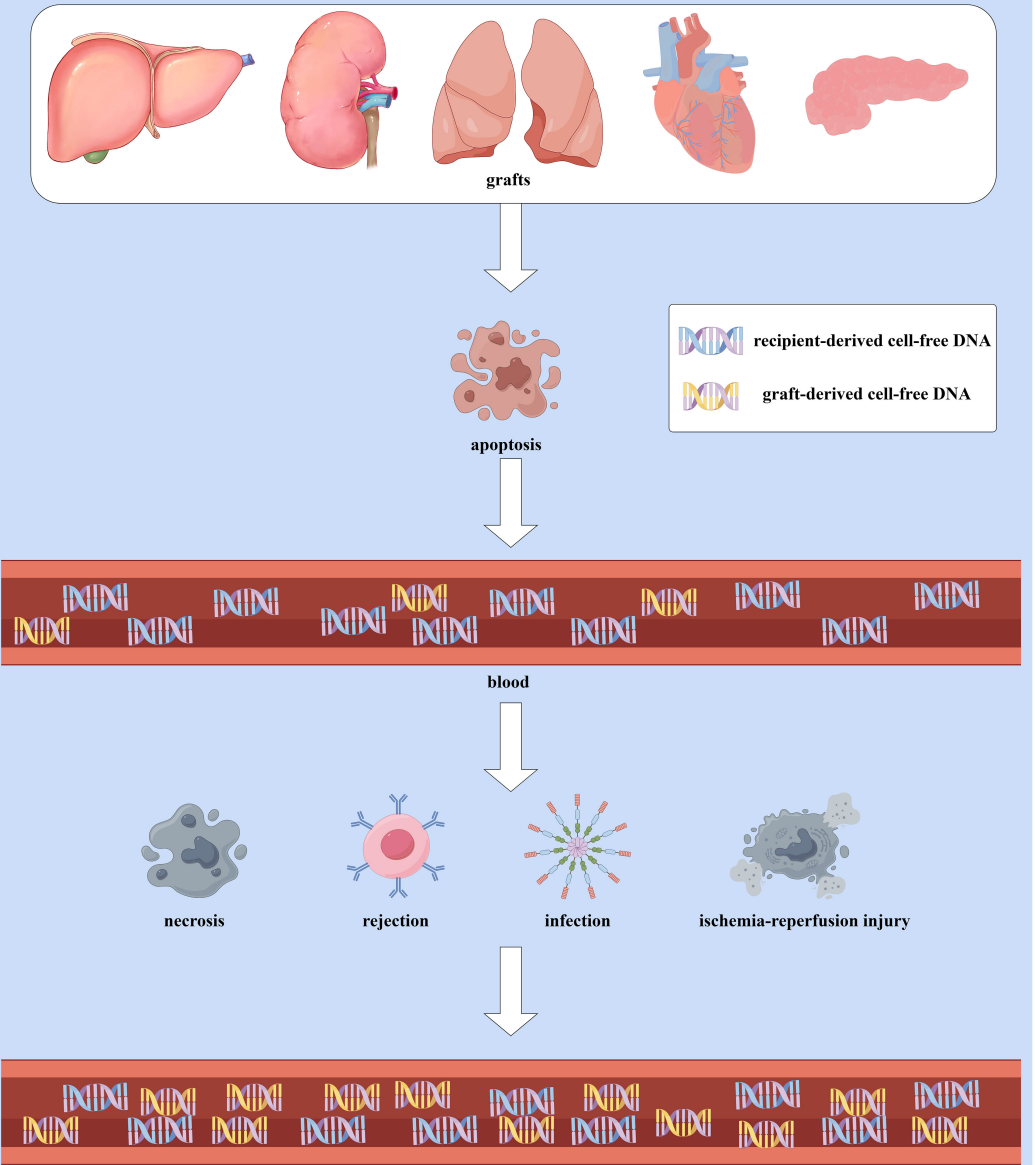
The Production and elevation of gd-cfDNA: When the graft is stable, gd-cfDNA in the plasma primarily originates from the apoptosis of graft cells, constituting only a small portion of the total cfDNA in the recipient’s blood and is shorter in length compared to recipient-derived cfDNA. However, in cases of ischemia-reperfusion injury, rejection, infection, or other causes leading to graft cell necrosis, more gd-cfDNA will released into the recipient’s bloodstream, resulting in an increase in quantification of gd-cfDNA. (By Figdraw).

In the cell nucleus, DNA wraps around histones to form nucleosomes. During cell damage, apoptosis, or necrosis, chromosomal DNA undergoes degradation and release. Initially, DNA is cleaved into large fragments (50–300 kb), followed by further degradation, resulting in the release of smaller DNA fragments (180–200 bp) and nucleosomes into the blood. These DNA fragments are eventually cleared in the liver and kidneys (6–8). The half-life of these DNA fragments ranges from approximately 15 to 90 minutes (9). These double-stranded DNA fragments circulating in the plasma are called cfDNA, also known as circulating free DNA or extracellular DNA. The discovery of cfDNA dates back to 1948 (10). As a reliable “liquid biopsy” method, cfDNA has been widely used in fields such as prenatal diagnosis and cancer monitoring (11, 12).

gd-cfDNA is a specific subcategory of cfDNA and refers to the cfDNA derived from graft cells circulating in the recipient’s plasma after transplantation. The majority of cfDNA in the recipient’s blood (over 95%) originates from the recipient’s apoptotic hematopoietic cells (13). Compared to hematopoietic-derived DNA (with a peak length of approximately 166 bp), non-hematopoietic-derived DNA is shorter, with gd-cfDNA primarily ranging from 105 to 145 bp (14). The level of graft-derived cell-free DNA (gd-cfDNA) is typically quantified in two ways: relative quantification as gd-cfDNA(%) and absolute quantification as gd-cfDNA(cp/mL), which refers to the percentage of gd-cfDNA relative to total circulating cfDNA and the number of gd-cfDNA copies per milliliter of serum, respectively (15). When the graft is stable, the amount of gd-cfDNA released from apoptotic graft cells constitutes only a tiny fraction of the total cfDNA in the recipient’s blood, and it is shorter in length than recipient-derived cfDNA (13, 14, 16). When the graft experiences ischemia-reperfusion injury, rejection, or infection, resulting in graft cell necrosis, varying amounts of gd-cfDNA are released into the recipient’s plasma. (**Figure 1**). The extent of gd-cfDNA elevation correlates with the type and severity of graft injury, making Changes in gd-cfDNA levels a dynamic indicator for monitoring graft injury (17). Since gd-cfDNA has an overall negative charge, it cannot be filtered by the glomerulus (18); it is typically measured in blood. But unlike other organ transplants, in kidney transplantation, if there is tubulitis or interstitial inflammation, gd-cfDNA can also be excreted through the kidneys, leading to elevated gd-cfDNA levels in urine (8, 19). Thus, urinary gd-cfDNA levels can also reflect the status of the transplanted kidney. Overall, by detecting gd-cfDNA in blood or urine samples, gd-cfDNA can dynamically and early reflect the graft status, providing clinicians with a basis for decision-making (20).

## 3 Detection methods of gd-cfDNA

In 1998, Lo et al. (21) identified Y chromosome-specific genes from donors in female recipients using polymerase chain reaction (PCR). However, at that time, the detection method was limited to specific cases where the donor and recipient were of different sexes. Currently, gd-cfDNA detection methods can be categorized into targeted and random approaches (**Table 1**). The quantitative detection of gd-cfDNA primarily relies on genetic markers, typically single nucleotide polymorphisms (SNPs), to differentiate between donor and recipient alleles (28). The development of quantitative techniques has provided diverse options for clinical practice. Real-time quantitative PCR (qPCR), as an early commonly used technique, is characterized by its simplicity, low cost, and rapid processing time, though it has relatively lower sensitivity and specificity (22, 29). Droplet digital PCR (ddPCR) has high sensitivity and accuracy compared to qPCR, but it may require more expensive reagents and equipment, and data analysis and interpretation may take longer (22, 30). Next-generation sequencing (NGS) technology, with its ability to sequence thousands of targets simultaneously, provides high sensitivity and accuracy for detecting complex genetic variations and unknown sequences (31). This capability makes NGS particularly advantageous in multiple transplants, multi-organ transplants, and monitoring rejection and infection (24, 32, 33). Despite the challenges of cost, technical complexity, and analysis time associated with NGS (24, 34, 35), its capability in gd-cfDNA monitoring cannot be overlooked. The turnaround time for ddPCR is shorter, with results typically available in one working day, whereas NGS requires 2–3 working days (36). These methods can provide relative quantification in relation to a calibrator, but only ddPCR can achieve absolute quantification (37).

**Table 1:** Comparison of targeted approach and random approach.

Approach	Method	Technology	Measurement	Genotyping required
Targeted	Targeted approaches in ddPCR or targeted NGS use preselected SNPs with high minor allele frequency, increasing the chance of donor-recipient allele differences. If the recipient is homozygous for an SNP, any alternative allele fragment is from the graft, so only a few SNPs are needed, and donor genotyping isn’t required (22, 23).	PCR-based with ddPCR read-out	TheraSure (22)	Recipient
		PCR-based with NGS read-out	AlloSure (24) or Prospera (25)	Not required
Random	Random approaches use adapter ligation and NGS to sequence dsDNA fragments with SNP markers directly from plasma. In this method, the donor and recipient are genotyped, often with SNP-chip technology, to assign sequencing reads. Alternatively, the gd-cfDNA fraction can be estimated using a statistical model that considers population allele frequency, donor-recipient kinship, and sequencing/genotyping error rates, eliminating the need for donor genotyping (23, 26).	Ligation-based	Transplant Rejection Assessment using gd-cfDNA (TRAC) (27)	Recipient or Donor and Recipient

## 4 Comparison of gd-cfDNA with common monitoring indicators

Histopathological biopsy remains the current gold standard for organ transplant monitoring and diagnosis, but it requires operation by specialized medical personnel (38, 39). Moreover, approximately 25% of kidney biopsy samples lack sufficient glomeruli, especially when using finer biopsy needles, which may increase the likelihood of requiring repeated punctures and thus elevate unnecessary complications risk (40). Additionally, biopsy sampling captures only a few glomeruli and a limited number of renal tubules from a localized area, leading to sampling errors and an incomplete understanding of the overall condition of the kidney. Studies have shown that 43% of clinically indicated biopsies and 65% of protocol biopsies are unnecessary (41). Some biomarkers used to monitor graft function, such as plasma creatinine, can measure glomerular function but are not sensitive indicators of graft injury. By the time complications like rejection lead to a significant rise in plasma creatinine, substantial tissue damage may have already occurred in the transplanted kidney (42). Similarly, other commonly used indicators for monitoring organ function also have some limitations. (**Table 2**).

**Table 2:** Limitations of common monitoring indicators and findings of gd-cfDNA research.

Organs	Indicator	Limitations	Findings of gd-cfDNA
Liver	Biopsy	Risk of bleeding, infection, sepsis, and bile leak (43)	Early predictive value: peaked 1–2 weeks before liver biopsy diagnosis of AR (44).
	Liver function tests	Inability to accurately assess rejection and infection (45, 46); Liver enzyme half-lives are long, with aspartate aminotransferase (AST) at 17 hours and alanine aminotransferase (ALT) at 47 hours, resulting in slow clearance and lag (47)	Short half-life and increased 4–6 days before aminotransferase elevation (48, 49). The ROC-AUC for diagnosing acute rejection (AR) was 0.99, outperforming alanine aminotransferase (0.86), alkaline phosphatase (0.66), γ-glutamyl transferase (0.80), and bilirubin (0.35) (50).
Kidney	Biopsy	High incidence of complications such as hematuria, perirenal hematoma, and arteriovenous fistula (51)	Provides more comprehensive graft information when combined with Banff scoring (52). Increased 30 days before histological changes (27).
	Serum creatinine, proteinuria, and eGFR	Low specificity and lag in distinguishing rejection from infection (53)	ROC-AUC for rejections was 0.80, significantly higher than serum creatinine (0.50) and eGFR (0.74) (25, 54).
Lung	Biopsy	Risk of bleeding, pneumothorax, and mediastinal emphysema (55)	Increased an average of 2.8 months before biopsy and lung function tests showed abnormalities (56).
	Bronchoscopy	Due to complications such as accidental bleeding and injury, radiological diagnosis of graft injury is often relied upon (57)	High sensitivity and specificity for detecting clinically asymptomatic graft injury (31).
Heart	Biopsy	Risk of cardiac perforation, valvular damage, and nerve injury (39)	Detected graft injury 0.5–3.2 months before biopsy-confirmed rejection (58).
	Left ventricular ejection fraction	Typically occurs at a late stage, with low sensitivity for monitoring rejection (59)	Detects all rejection types before clinical dysfunction occurs (35).
	Troponin, B-type Natriuretic Peptide, C-Reactive Protein, etc.	Limited monitoring value, no longer used for rejection screening (60–62)	High sensitivity and ability to distinguish rejection types (63).
Pancreas	Biopsy	Risk of infection, elevated blood amylase, and pancreatic fluid leakage (64)	It can differentiate between pancreatic rejection and pancreatitis (65).
	Secretory function indicators, such as amylase, HbA1c, C-peptide, etc.	Low specificity; pancreatic exocrine function may be normal, and elevation may not accurately reflect graft status (65)	ROC-AUC for diagnosing acute pancreatic rejection (0.89) significantly outperformed lipase (0.74) and amylase (0.46) (66).

ROC-AUC, Receiver Operating Characteristic - Area Under the Curve; DSA, Donor-specific antibody.

Donor-specific antibody (DSA) is a crucial biomarker for predicting the development of antibody-mediated rejection (AMR) and assessing graft survival (67). However, limitations in DSA detection—such as variability in detection methods, sensitivity, and clinical significance standards—restrict its role in rejection (68, 69). Although increasing sensitivity of DSA detection assays, a significant proportion of patients with the same histologic picture of ABMR, do not have detectable circulating DSA (70, 71). A recent kidney transplant study indicated that gd-cfDNA might be a better predictor of AMR than DSA. In that study, DSA was frequently negative in both molecular (56%) and histologic (51%) AMR. In AMR, gd-cfDNA(%)≥1.0 was more frequent (75%) than DSA positivity (44%). Moreover, all AMR patients, DSA-positive or DSA-negative, showed elevated gd-cfDNA levels (mean 1.88% vs. 0.32% in the non-rejection group) (72). Some studies have demonstrated a correlation between elevated gd-cfDNA levels and the occurrence of DSA, allowing for the prediction of DSA development. In heart transplantation, gd-cfDNA levels were significantly higher in patients who experienced *De novo* donor-specific antibodies (dnDSA) compared to those who did not (0.34% vs. 0.06%) and were elevated a median of 20 days before dnDSA was detected (73). In kidney transplant recipients, 40% (17/42) of patients with gd-cfDNA ≥0.5% developed dnDSA, compared to only 2.7% (1/37) of patients with gd-cfDNA <0.5%, showing a significant difference (74). Kidney transplant recipients with gd-cfDNA levels of 0.5% or higher have nearly a threefold increased risk of developing dnDSA, with levels rising a median of 91 days before the identification of DSA (interquartile range, 30–125 days) (54). Similarly, in heart transplantation, among 613 DSA samples paired with gd-cfDNA levels, gd-cfDNA greater than 0.15% was associated with a fourfold increase in the incidence of dnDSA in the first year post-transplantation (75). However, research on the diagnostic performance of gd-cfDNA and DSA in rejection remains limited, and further studies are needed to confirm their roles.

gd-cfDNA is directly derived from the graft and has a short half-life, providing high sensitivity to tissue damage. The minimally invasive nature of blood or urine sampling for gd-cfDNA testing further adds to its advantages (5, 72). From an economic perspective, gd-cfDNA monitoring also offers substantial benefits. The estimated total cost of a kidney biopsy is approximately $3,931 (76), and the average cost of an endomyocardial biopsy (EMB) after heart transplantation is as high as $7,918 (77). If biopsy-related complications occur (such as hospitalization and hematoma evacuation), additional costs can increase by an average of $10,743 (78). In comparison, the cost of ddPCR for the first year is about $4,012 (including testing on days 7 and 14 post-transplant, monthly testing for the first 6 months, and quarterly testing thereafter). In subsequent years, quarterly testing with ddPCR costs around $1,604 (53). The cost difference and risk considerations make gd-cfDNA a valuable non-invasive monitoring method in managing organ transplant recipients.

It is important to note that gd-cfDNA testing cannot yet fully replace pathological biopsy as the gold standard for diagnosing rejection; it serves as a complementary test or routine monitoring tool. Compared to creatinine, gd-cfDNA more effectively guides the timing and necessity of clinical biopsies, significantly reducing the number of biopsies and associated risks (37).

## 5 Baseline levels of gd-cfDNA

The baseline level of gd-cfDNA is the stable value of gd-cfDNA observed in the recipient’s blood in the early post-transplant period after the concentration of gd-cfDNA has declined from the initial peak caused by surgical trauma and ischaemia-reperfusion injury. It is critical for monitoring the health of the graft, as deviations from this baseline may be indicative of graft injury, such as rejection (22, 79). The baseline gd-cfDNA levels are influenced by several factors, including the type of transplanted organ, the recipient’s immune response, and the extent of surgical trauma during transplantation. The baseline levels vary by organ type. The speed at which elevated gd-cfDNA levels return to baseline also varies among individuals and types of transplanted organs. **Table 3** summarizes the baseline levels of gd-cfDNA found in current studies after various organ transplants and the recovery Times to baseline levels (22, 80, 81). Pancreas transplantation has higher risks for undesirable immune response and complications compared with other types of organ transplantation, which may contribute to the relatively slow return to gd-cfDNA baseline levels after pancreas transplantation, but more research is needed to discover the exact reasons for this (62, 63). Furthermore, baseline levels of gd-cfDNA in liver and lung transplants are slightly higher than in kidney and heart transplants, possibly due to difference in the number of cells in the graft tissue (22). In recipients of stable bilateral lung transplants, the median gd-cfDNA level is higher than those of single lung transplants (0.46% vs. 0.15%) (82).

**Table 3:** Baseline levels of gd-cfDNA and recovery times for various organs.

Organs	Baseline levels	Recovery times
Liver	3.3%-5.0%	1-2 weeks
Kidney	0.3%-1.2%	1-2 weeks
Lung	1.0%-3.0%	2-3 weeks
Heart	0.1%-0.5%	1-2 weeks
Pancreas	0.1%-1.0%	1 month

## 6 The role of gd-cfDNA in organ transplantation

### 6.1 gd-cfDNA and rejection

Rejection remains a severe complication affecting graft function and recipient survival post-transplantation. Timely diagnosis and treatment are vital to ensuring successful transplantation and long-term survival (83). During graft rejection, gd-cfDNA (%) levels increase significantly (84), and this rise occurs earlier than other indicators, such as serum creatinine and transaminases (85).

Studies indicate that for every 1% increase in gd-cfDNA levels, the risk of rejection increases 3.3-fold, with an overall rejection risk ratio of 1.89 (54). gd-cfDNA levels are significantly positively correlated with the type and severity of graft injury and Banff rejection scorings (23, 86–89). For instance, in kidney transplantation, the median gd-cfDNA in the non-rejection group is 0.3%, while in AMR, it is 2.9%, and in T-cell-mediated rejection (TCMR), it is 1.2%, with the highest levels observed in AMR (5). This may be due to AMR being the most severe and destructive form of immune-mediated graft injury, leading to more cell necrosis and the release of gd-cfDNA (90). In reflecting the severity of rejection, gd-cfDNA levels post-liver transplantation are 9.1%, 12.1%, and 28.6% in mild, moderate, and severe acute rejection (AR), respectively, significantly higher than the 0.16% in the non-rejection group and differences in gd-cfDNA levels exist among AR patients of varying severity (91). gd-cfDNA as a non-invasive marker for allograft rejection monitoring has the potential to be a valuable non-invasive marker for allograft rejection monitoring and has already been utilized by the International Society for Heart and Lung Transplantation (ISHLT) for graft rejection monitoring (92–94).

To better use gd-cfDNA for monitoring and identifying rejection post-organ transplantation, different studies have set varying gd-cfDNA thresholds to analyze diagnostic efficacy for graft injury (**Table 4**). In patients with stable graft status during the first year after liver transplantation, a 10% threshold demonstrated higher diagnostic efficacy for rejection than traditional liver function tests (LFTs) conducted on the same day (48). In kidney transplantation, 0.5%–1.0% thresholds showed good diagnostic performance for rejection, outperforming serum creatinine and eGFR (25, 53, 54). After lung transplantation, gd-cfDNA levels below 1.0% help exclude AR (102). In heart transplantation, when gd-cfDNA does not exceed 0.25%, there is a high confidence that rejection has not occurred, and this threshold is widely applied clinically (63). There is a lack of research on gd-cfDNA monitoring after pancreas-only transplantation. However, after simultaneous pancreas-kidney transplantation (SPK), a gd-cfDNA threshold of 70 cp/mL has been effectively used to detect early pancreatic graft rejection (66).

**Table 4:** Diagnostic efficacy of different gd-cfDNA thresholds in graft injury.

Organs	First author, Year	Reference	Sample types	Injury types	Thresholds	ROC-AUC	Sensitivity	Specificity	PPV	NPV
Kidney	Sigdel, 2013	(95)	Urine	AR	3 cp/μg creatinine	0.80	81.00%	75.00%	NA	NA
	Oellerich, 2019	(37)	Plasma	Rejection	0.43%	0.73	73.00%	69.00%	12.00%	98.00%
	Bu, 2022	(54)	Plasma	Rejection	0.50%	0.80	78.00%	71.00%	50.00%	90.00%
	Huang, 2018	(96)	Plasma	Rejection	0.74%	0.71	79.40%	72.40%	77.10%	75.00%
	Huang, 2018	(96)	Plasma	AMR	0.74%	0.82	100.00%	71.80%	68.60%	100.00%
	Bloom, 2017	(5)	Plasma	AR	1.00%	0.74	59.00%	85.00%	61.00%	84.00%
	Bloom, 2017	(5)	Plasma	AMR	1.00%	0.87	81.00%	83.00%	44.00%	96.00%
	Sigdel, 2018	(25)	Plasma	AR	1.00%	0.87	88.70%	72.60%	51.90%	95.10%
	Graver, 2023	(27)	Plasma	AR	0.5%-1%	NA	50%-100%	69%-96%	12%-77%	75%-98%
	Oellerich, 2021	(53)	Plasma	Rejection	0.74%-1.0%	0.74	80.00%	76.00%	56.00%	90.00%
	Whitlam, 2019	(97)	Plasma	AMR	21 cp/mL	0.92	90%	88%	60%	98%
	Oellerich, 2019	(37)	Plasma	Rejection	52 cp/mL	0.83	73.00%	73.00%	13.00%	98.00%
Liver	Levitsky, 2022	(85)	Plasma	AR	5.30%	0.95	87.00%	NA	NA	100.00%
	Schütz, 2017	(48)	Plasma	AR	10.00%	0.97	90.30%	92.90%	NA	NA
	Fernández-Galán, 2022	(44)	Plasma	AR	13.80%	0.77	85.70%	63.30%	35.30%	95.50%
	Levitsky, 2022	(85)	Plasma	AR	20.40%	0.71	66.70%	NA	NA	87.80%
	Zhao, 2021	(98)	Plasma	Pediatric Rejection	28.70%	0.88	72.70%	94.70%	80.00%	92.30%
	Zhao, 2021	(98)	Plasma	Pediatric Rejection	2076 cp/mL	0.84	81.80%	81.90%	56.20%	93.90%
	Cox, 2022	(91)	Plasma	TCMR	33.50%	0.73	NA	97.00%	NA	86.10%
	Goh, 2019	(50)	Plasma	AR	898 cp/mL	0.99	83.30%	100.00%	100.00%	87.70%
Lung	Agbor-Enoh, 2018	(56)	Plasma	Rejection	0.50%	0.80	78.00%	71.00%	50.00%	90.00%
	Jang, 2021	(79)	Plasma	AR	0.50%	0.89	95.00%	65.00%	85.63%	92.92%
	Khush, 2021	(99)	Plasma	AR	0.85%	0.67	55.60%	74.80%	70.00%	63.04%
	Sayah, 2020	(100)	Plasma	AR	0.87%	0.72	73.10%	52.90%	60.78%	65.71%
	DeVlaminck, 2015	(101)	Plasma	AR	1.00%	0.90	100.00%	73.00%	83.80%	88.10%
	Keller, 2022	(102)	Plasma	AR	1.00%	0.82	73.90%	87.70%	43.40%	96.50%
	Keller, 2022	(102)	Plasma	AR	1.10%	0.86	78.00%	83.00%	81.88%	79.14%
	Sorbini, 2022	(103)	Plasma	AR	1.25%	0.87	80.70%	73.00%	75.47%	80.00%
	Pedini, 2023	(84)	Plasma	AR	1.72%	0.80	NA	NA	75.00%	91.40%
	Trindade, 2023	(104)	Plasma	ALAD	0.85%-1%	0.87	87.00%	78.00%	74.00%	89.00%
Heart	Khush, 2021	(105)	Plasma	AR	0.15%	NA	55.90%	71.50%	7.80%	97.40%
	Kim, 2022	(106)	Plasma	AR	0.15%	0.86	78.50%	76.90%	97.30%	25.10%
	Khush, 2021	(105)	Plasma	AR	0.20%	0.64	44.10%	80.40%	8.90%	97.10%
	Agbor-Enoh, 2021	(58)	Plasma	AR	0.25%	0.92	NA	NA	NA	99.00%
	Kittleson, 2021	(63)	Plasma	AR	0.25%	0.92	81.00%	NA	NA	99.00%
	Holzhauser, 2023	(3)	Plasma	AR	0.25%	0.92	81.00%	85.00%	NA	99.20%
	Borkowski, 2024	(107)	Plasma	AR	0.25%	NA	NA	NA	NA	99.00%

AR, Acute Rejection; AMR, Antibody Mediated Rejection; TCMR, T-cell-mediated rejection; ALAD, Acute lung allograft dysfunction; NA, not available; ROC-AUC, Receiver Operating Characteristic - Area Under the Curve; PPV, Positive predictive value; NPV, Negative predictive value.

It is important to note that plasma gd-cfDNA levels are relatively low in early Banff 1A and borderline TCMR in kidney transplants. The median gd-cfDNA level in TCMR patients is 0.7%, but it is only 0.20% in borderline TCMR patients (Banff t1/i1), even lower than the 0.23% in the non-rejection group, leading to poor diagnostic accuracy (54). This may be because borderline and Type I (IA and IB) TCMR primarily manifest as tubulitis and interstitial inflammation, damaging tubular epithelial cells, which increases urinary gd-cfDNA levels but has a smaller effect on plasma gd-cfDNA levels (68, 108). Therefore, it is recommended to simultaneously test plasma and urinary gd-cfDNA in kidney transplant patients for a comprehensive assessment.

In lung transplant recipients, gd-cfDNA (%) median levels significantly increase in patients with AR (12.0%), higher than in stable patients (1.1%). After the resolution of rejection, gd-cfDNA levels decrease to the level in the stable group (57). Other study has also shown that after early aggressive treatment of AR, gd-cfDNA levels gradually decline and return to lower levels (<0.5%), providing crucial information for monitoring the efficacy of rejection treatment (109).

### 6.2 gd-cfDNA and other types of graft injury

gd-cfDNA is not a specific marker for rejection and is also associated with various types of graft injury, though the degree of elevation varies, which can help in differentiation (110) (**Table 5**). Furthermore, not all types of infections result in elevated gd-cfDNA levels. For instance, cytomegalovirus infections (e.g., cytomegalovirus-related hepatitis) do not cause direct graft injury and therefore do not lead to an increase in gd-cfDNA levels (33).

**Table 5:** Relationship between different types of graft injury and gd-cfDNA.

Organs	Injury types	Findings
Liver	Hepatitis C virus infection	Compared to 88 recipients with stable graft function (median gd-cfDNA of 3.3%), 17 recipients infected with hepatitis C virus (5.9%) showed a slight increase, which was lower than the 17 patients in the AR group (29.6%), with statistically significant differences between each group (48).
	Graft-versus-host disease (GVHD)	GVHD lacks sensitive and specific biomarkers for assessing the immune status of solid organ recipients; 97% of granulocytes in the blood were observed to be of donor origin in a patient who developed GVHD, with a higher proportion of T cells and B cells, and significantly elevated gd-cfDNA levels (39.8%). After aggressive treatment, the levels of gd-cfDNA (%) and donor-derived immune cells decreased, indicating that gd-cfDNA might be a promising biomarker for monitoring GVHD and treatment efficacy (111).
	Infection and drug-induced liver injury	All 49 recipients in the study developed complications. Among the 11 patients with rejection, the median gd-cfDNA was 41.7%, 16.6% in the 10 patients with EBV infection, 11.2% in the 22 patients with CMV infection, and 7.8% in the 6 patients with drug-induced liver injury. There was a significant difference between the rejection group and the other groups, but no statistical difference among the other three groups; gd-cfDNA can distinguish infection and drug-induced liver injury from rejection (98).
Kidney	Fibrosis/tubular atrophy and acute tubular necrosis	Among 189 kidney transplant patients, the median gd-cfDNA was 0.29% in 83 stable patients, 0.57% in 15 patients with rejection, 0.46% in 24 patients with interstitial fibrosis/tubular atrophy (IF/TA), and 0.46% in 29 patients with acute tubular necrosis (ATN). There were significant differences between the three groups with complications and the stable group; IF/TA also showed significant differences compared to the other two groups. Therefore, elevated gd-cfDNA is associated not only with rejection but also with IF/TA and ATN (37).
	Acute tubular necrosis and acute pyelonephritis	Increases in gd-cfDNA (%) (>0.88%) were significantly associated with acute tubular necrosis and acute pyelonephritis; however, there was no correlation with BKV or CMV infection, symptomatic lower urinary tract infections, fluid retention, prerenal acute kidney injury, or serum creatinine elevation due to calcineurin inhibitor use (112).
	BK polyomavirus-associated nephropathy (BKPyVAN)	The urinary gd-cfDNA concentration had a true positive rate of 95.2% for diagnosing BKPyVAN, significantly higher than the 71.4% and 33.3% for urinary and plasma BKV-DNA loads, respectively; suggesting that urinary gd-cfDNA is better than urinary/plasma BKV viral load for diagnosing BKPyVAN (110).
Lung	Primary graft dysfunction (PGD)	There is an association between gd-cfDNA and the occurrence and severity of PGD: on post-transplant day 3, gd-cfDNA (%) in PGD patients was significantly higher than in non-PGD patients (median: 12.2% vs 8.5%) (113).
	Pulmonary infection and reduced oxygenation	The median gd-cfDNA (12.0%) in the acute rejection group showed a significant difference compared to the infection group (4.2%) and the stable group (1.1%), which helps distinguish rejection from infection, but the difference between the infection group and the stable group was not statistically significant; the gd-cfDNA level was negatively correlated with oxygenation levels (PaO2/FiO2 ratio) (57)
	Respiratory viral infection (RVI)	An increase in gd-cfDNA (≥1%) in 7 days after RVI was closely associated with the development of CLAD, decline in lung function, and increased risk of graft failure (114).
	Chronic lung allograft dysfunction (CLAD)	Early post-transplant levels of gd-cfDNA in PGD patients were higher, and those who developed CLAD had gd-cfDNA levels about twice as high as those who did not develop CLAD (median: 22.4% vs 9.9%), with the difference being statistically significant (113).
Heart	Cardiac allograft vasculopathy (CAV)	Compared to samples without CAV, recipients with CAV had significantly elevated gd-cfDNA levels (mean 0.47% vs 0.09%), with a statistically significant difference (115).
Pancreas	Pancreatitis	In a study of 46 pancreatic transplants, the median gd-cfDNA was 2.25% in 13 patients with rejection, 0.36% in 2 patients with pancreatitis, and 0.18% in the stable group; there was a significant difference in gd-cfDNA levels between the rejection/pancreatitis group and the stable group (65).

gd-cfCDNA, graft-derived cell-free DNA; AR, Acute rejection; GVHD, Graft-versus-host disease; CMV, Cytomegalovirus; EBV, Epstein-Barr Virus; BKPyVAN, BK polyomavirus-associated nephropathy; IF/TA, Interstitial fibrosis/tubular atrophy; ATN, Acute tubular necrosis; PGD, Primary graft dysfunction; RVI, Respiratory viral infection; CLAD, Chronic lung allograft dysfunction; CAV, Cardiac allograft vasculopathy.

BK polyomavirus-associated nephropathy (BKPyVAN) is also a severe complication that can lead to graft dysfunction and loss in kidney transplantation. BKPyVAN is the most severe stage of BK virus infection, causing the death of graft kidney cells and releasing high levels of gd-cfDNA (116). However, BKPyVAN shares features with TCMR, especially Banff IA and IB TCMR, both characterized by tubulointerstitial inflammation and graft dysfunction (68, 117). The standard diagnosis of BKPyVAN is through histopathological detection of Simian virus 40 (SV40) in kidney biopsy tissue (118). However, due to the focal nature of BKPyVAN lesions, there is a high false-negative rate in biopsies (119), making it difficult to distinguish between the two when SV40 staining is negative. Since the treatment principles for BKPyVAN and TCMR are entirely different, there is an intense need for more precise diagnostic techniques. Fortunately, elevated urinary gd-cfDNA levels are more significantly associated with BKPyVAN, aiding in differentiation (110, 120). Although BKPyVAN and Type I TCMR have similar histological features, biopsy-confirmed BKPyVAN recipients have significantly higher urinary gd-cfDNA (%) and concentrations than those with Type I TCMR (median 68.4% vs. 55.3% and 10.4 ng/mL vs. 6.1 ng/mL, respectively) (117). This might be because BKPyVAN primarily infects tubular epithelial cells, leading to the kidney cell lysis and the release of gd-cfDNA into the tubular lumen, which is then excreted in the urine (121, 122). Whereas the TCMR mainly results in inflammatory cell infiltration rather than cell lysis (68). Shen et al. (117) identified a urinary gd-cfDNA concentration threshold of 7.81 ng/mL, which may effectively distinguish confirmed BKPyVAN from Type I TCMR.

### 6.3 Monitoring immunosuppressive therapy with gd-cfDNA

Organ transplant recipients require long-term immunosuppression to prevent immune rejection (1). The application of gd-cfDNA in monitoring the effectiveness of immunosuppressive therapy is gaining increasing attention, particularly in detecting rejection due to inadequate immunosuppression, where gd-cfDNA outperforms traditional therapeutic drug monitoring (53). Given the high variability in individual sensitivity to immunosuppressive drugs, maintaining an immunosuppressive therapeutic window through drug concentration monitoring may not be suitable for every patient. Moreover, there is a significant difference in drug concentrations between blood and lymphocytes for every patient, meaning that immunosuppressant levels may not accurately reflect the drug’s impact on immune cells (123–125). gd-cfDNA levels can predict damage before the onset of severe AR symptoms; if immunosuppressant drug levels are below the therapeutic range at this time, it indicates a need to increase the dosage (85).

Oellerich et al. (126) simultaneously measured gd-cfDNA levels and tacrolimus trough concentrations in liver transplant patients, finding that when drug concentrations were in the therapeutic range and gd-cfDNA relative quantification was below 10%, graft function remained stable. Furthermore, when gd-cfDNA relative quantification was below 10% post-liver transplantation, the minimum effective tacrolimus trough concentration was 6.8 ng/mL (originally used 8 ng/mL), suggesting that combined monitoring of gd-cfDNA relative quantification could help reduce the drug dosage. Another study conducted a 180-day follow-up on a liver transplant recipient with hepatorenal syndrome. Due to the severe renal insufficiency and worsening symptoms experienced by this recipient, tacrolimus was switched to everolimus on postoperative day 84. When tacrolimus blood levels dropped to the lower end of the therapeutic range, gd-cfDNA (%) rapidly increased to 66%. Nevertheless, as everolimus blood levels reached the therapeutic range, gd-cfDNA (%) quickly decreased and remained below 5%, indicating that gd-cfDNA (%) correlates with the concentration of immunosuppressants in the appropriate therapeutic window (20).

In a kidney transplant study, 6 months of gd-cfDNA monitoring was performed after adjusting the immunosuppressant mycophenolic acid (MPA). Among 17 recipients in the low-risk group (gd-cfDNA <1%), no rejection occurred after MPA dose reduction; however, in 4 patients in the high-risk group (gd-cfDNA ≥1%) whose MPA dosage remained unchanged, 2 developed graft dysfunction, and 1 experienced graft loss (127). Continuous monitoring and dynamic changes in gd-cfDNA can guide clinicians in making more precise decisions when adjusting immunosuppressant doses, contributing to personalized treatment in immunosuppressive therapy and demonstrating significant potential in improving the management of organ transplant recipients (27, 85).

### 6.4 Prognostic prediction with gd-cfDNA

Early dynamic changes in gd-cfDNA post-transplantation can reflect the recovery of organ function and provide information related to long-term prognosis. In kidney transplantation, gd-cfDNA levels below 0.5% on postoperative day 7 are considered normal recovery, whereas patients with levels equal to or greater than 0.5% had a median dialysis time of 13.50 days post-transplantation. The median gd-cfDNA level in patients with delayed graft function (fDGF) in 24 hours reached 7.20%, while it was 2.70% in those without functional DGF. Patients whose gd-cfDNA decreased to below 0.5% in 7 days postoperatively had a higher 7-year expected graft survival rate compared to those whose gd-cfDNA levels remained at or above 0.5% after 7 days (79.5% ± 16.8% vs. 67.7% ± 24.1%) (128). Patients with gd-cfDNA ≥0.5% had a doubling increased risk of a 25% decline in eGFR in three years post-transplantation (54).

Compared to patients with low and moderate gd-cfDNA levels (low, medium, and high gd-cfDNA levels were 0.7%, 1.6%, and 3.6%), those with high gd-cfDNA levels (%) after lung transplantation had a 6.6-fold increased risk of graft failure, with progression to CLAD or death (31). Additionally, plasma gd-cfDNA levels negatively correlated with oxygenation levels (PaO2/FiO2 ratio) immediately after and 72 hours post-lung transplantation, suggesting that increased gd-cfDNA levels may indicate declining oxygenation capacity of the transplanted lung (57).

Approximately 50% of heart transplant recipients develop cardiac allograft vasculopathy (CAV) 10 years post-transplantation, a major obstacle to long-term survival typically detected via selective coronary angiography, which carries significant risk (129). An increase in gd-cfDNA levels to ≥0.12% two years post-transplantation is significantly associated with the occurrence of CAV (130). Early evaluation of such patients helps in personalizing the understanding of CAV risk. Furthermore, higher gd-cfDNA levels post-heart transplantation are associated with a composite endpoint of death, re-transplantation, hemodynamic compromise, or graft dysfunction in three years (131). Recipients with left ventricular ejection fraction (LVEF) below 40% had significantly higher gd-cfDNA (%) than those with LVEF of 40% or above (0.46% vs. 0.04%) (106).

gd-cfDNA monitoring can dynamically reflect graft status, helping doctors identify high-risk patients for potential graft dysfunction or early decline, allowing timely intervention or treatment. gd-cfDNA shows potential and application value in prognosis prediction post-organ transplantation, though further research is needed to verify its clinical accuracy and reliability.

## 7 Advantages

The main advantages of gd-cfDNA can be summarized as follows:

1. Non-invasive Monitoring: gd-cfDNA offers a more frequent and less invasive monitoring method than biopsy, reducing patient discomfort and risk.
2. Early Diagnosis, High Sensitivity, and Dynamic Monitoring: With a shorter half-life, gd-cfDNA demonstrates higher sensitivity. gd-cfDNA can provide early warnings before graft injury, such as rejection, enabling early diagnosis and timely treatment. The more severe the injury, the higher the gd-cfDNA levels (132).
3. Immunosuppressive Efficacy Allows for Personalized Treatment: Due to its short half-life, gd-cfDNA allows for longitudinal monitoring of disease progression, providing near real-time indications of organ injury (133). Dynamic monitoring of gd-cfDNA levels enables safer and more effective use of immunosuppressants, dose adjustments, and personalized treatment, improving the long-term survival quality of organ transplant recipients (20).
4. Predicting Transplant Outcomes: Elevated gd-cfDNA levels post-transplantation are associated with poor graft function recovery, graft injury, and inadequate immunosuppression, helping to predict long-term transplant outcomes (134).

## 8 Limitations

1. Limited Specificity for Injury Types: gd-cfDNA is highly sensitive in reflecting graft cell damage and death; however, its ability to distinguish the types of injuries is currently limited. It is not a specific marker for any type of injury but is observed in various graft injuries. Though studies have indicated that gd-cfDNA levels are higher in AR recipients compared to other pathological states caused by non-rejection factors (91). At present, gd-cfDNA may serve as a reliable marker for AMR, but its role in addressing Acute Cellular Rejection (ACR), chronic rejection, and other types of graft injuries has not yet been fully proven (135).
2. Uncertain sample stability: Most commercial assays require blood samples to be sent to external laboratories for analysis, potentially using different detection techniques, which may lead to inconsistencies in results and pricing. Additionally, the instability of cfDNA necessitates rapid and precise handling during sample transfer, transportation, and processing to avoid variations in results (24).
3. Uncertainty in Thresholds and Undefined Monitoring Frequency: There is variability in the diagnostic thresholds and monitoring frequency of gd-cfDNA across different studies, and no standardized guidelines currently exist. Further research is needed to determine the optimal monitoring frequency and thresholds, especially in the early post-transplant period when the risk of AR and infection is highest (54).
4. Global Disparities in Medical Resources: As an advanced medical technology, the accessibility of gd-cfDNA testing varies significantly across different countries and regions (136, 137).
5. Monitoring in Multi-Organ Transplants and Long-Term Monitoring: Due to the unique nature of multi-organ transplants, there is currently limited research on gd-cfDNA monitoring in these cases. Moreover, there is a lack of reports on routine graft monitoring beyond five years in clinically stable organ transplant recipients (3).

## 9 Discussion

As an emerging non-invasive biomarker, gd-cfDNA shows great potential in monitoring and prognostic assessment following organ transplantation. This is particularly significant for thoracic organs. Given the stringent size-matching requirements for thoracic organ transplants, the unique immunological characteristics post-lung transplantation, and the lack of extracorporeal life support options, long-term survival rates remain lower than other solid organ transplants (138, 139). Additionally, the incidence of AR in the first year post-lung transplantation is 26.6%, significantly higher than that of other organ transplants (140). Chronic lung allograft dysfunction (CLAD) occurs in approximately 50% of lung transplant recipients in five years, posing a major obstacle to long-term survival. Currently, there are no reliable predictive markers or effective preventive or therapeutic methods for CLAD (141), highlighting the urgent need for reliable graft monitoring tools. Currently, Primary graft dysfunction (PGD) and AR have been identified as strong independent risk factors for the development of CLAD (142, 143). The detection of gd-cfDNA enables the early identification of patients at risk for AR, PGD, and CLAD, facilitating timely therapeutic interventions that enhance the management of long-term complications and improve the prognosis of transplant recipients (141, 144).

Currently, research progress on gd-cfDNA varies across different organ fields. Some studies explored differences between graft injuries based on time trends, the extent of gd-cfDNA elevation, fragment length, and genomic composition. For example, in liver transplant research, the distribution and proportion of gd-cfDNA fragment sizes in plasma have been found to monitor post-transplant graft function (145). In lung transplantation, the combined approach of gd-cfDNA (%) and cfDNA fragment size is used to determine the occurrence and potential source of infection (84). Heart transplant studies have found that the genomic composition of DNA fragments differs between AMR and ACR, with AMR exhibiting a higher percentage of short fragments and higher guanine-cytosine content than ACR (58, 59). Transcriptome analysis of biopsy tissues has revealed that gd-cfDNA-associated gene expression patterns may be related to the type of rejection and response to treatment (146). These findings may help distinguish different types of injuries to better understand graft status monitoring.

Moreover, there is currently no consensus in clinical practice on whether to use gd-cfDNA (%) or gd-cfDNA (cp/mL) to optimize monitoring (147). The release of recipient cfDNA is influenced by factors such as immunosuppressants, age, and increased BMI (148). For instance, in two liver transplant studies, the median gd-cfDNA (%) in the AR group was 21.8% (91) and 41.9% (98), respectively. The former study population consisted of adult liver transplant recipients (median age 53.7 years), while the latter involved pediatric liver transplant recipients (median age 19.4 months). Schütz et al. (149) found that as the dose of immunosuppressants was reduced, the rate of leukocyte apoptosis decreased, leading to a decline in total cfDNA levels over time, while gd-cfDNA (%) correspondingly increased. However, the absolute quantification of gd-cfDNA remained stable during this period, indicating that the absolute quantification of gd-cfDNA may perform better than gd-cfDNA (%) in monitoring (37, 97). Nonetheless, the gd-cfDNA (%) assay was less sensitive to preanalytical variables and more advantageous in comparison among studies (150). In the field of lung transplantation, the concept of relative change value (RCV) in gd-cfDNA (%) has been proposed, suggesting that an increase of more than 73% from baseline may indicate pathological changes, thereby enhancing diagnostic performance (104).

gd-cfDNA can provide early warnings for rejection and various types of graft injuries with high sensitivity. However, gd-cfDNA monitoring cannot entirely replace biopsies; instead, it aids clinicians in more accurately identifying patients who genuinely need biopsies, reducing unnecessary invasive procedures and associated risks (151). As a single non-invasive test, gd-cfDNA has lower specificity for injury types. However, it can provide some information about the injury, which can be used to better determine the specific type of injury by combining it with other biomarkers or artificial intelligence techniques. For example, biopsy remains the gold standard for graft assessment, but relying solely on histological predictions has limitations; combining gd-cfDNA with Banff biopsy scores offers a more comprehensive assessment of graft injury and prognosis than biopsy features alone (52). Studies have shown that combining gd-cfDNA with DSA improves diagnostic accuracy for AMR compared to using either marker alone, and it significantly outperforms traditional graft function indicators (69, 72, 152). For instance, when predicting AMR, the AUC for gd-cfDNA (%) and DSA alone was 0.85 and 0.66, respectively, while the combined AUC was 0.88 (72). Some experienced heart transplant centers transition monitoring to a strategy that combines gene expression profiling (GEP) with gd-cfDNA in patients with stable grafts after 8 weeks post-transplant. GEP can assess the quiescent or activated state of the immune system, while gd-cfDNA monitoring serves as a specific marker for graft injury (151). Infection markers, such as procalcitonin (PCT), combined with gd-cfDNA, can enhance the ability to differentiate between AR and infection; during severe infections post-transplant, PCT is similarly elevated above the threshold when gd-cfDNA levels rise significantly, but PCT levels do not exceed threshold values in AR patients (153). gd-cfDNA monitoring combined with artificial intelligence leads to powerful predictive models, guiding clinical decisions and potentially better identifying high-risk patients (53, 98, 107). Additionally, combining gd-cfDNA with other testing methods, such as organ function tests, pathogen detection, and mRNA transcripts in the graft, may provide more comprehensive information on graft function, improve diagnostic efficiency, enable personalized post-transplant management, and ultimately enhance the quality of life and survival rates of transplant recipients (3).

In addressing the challenges of variable thresholds, undefined monitoring frequency, and global disparities in medical resources in the clinical application of gd-cfDNA, future research should focus on establishing standardized monitoring protocols, narrowing global healthcare gaps, and exploring new possibilities for gd-cfDNA. Through these efforts, gd-cfDNA has the potential to become a revolutionary monitoring tool in organ transplantation, providing more personalized and precise diagnostic information for transplant recipients, improving their quality of life, and increasing long-term survival rates.
